# Silent Retention: Endovascular Retrieval of a Guidewire Undetected for Three Months in an ESRD Patient: A Case Report

**DOI:** 10.1002/ccr3.72140

**Published:** 2026-02-28

**Authors:** Abdul Qadir Nawabi, Noor Ahmad Jamal, Wahidullah Lalzada, Ahmad Jamshed Rezaie, Aamna Israr

**Affiliations:** ^1^ Mellat Medical Institute Kabul Afghanistan; ^2^ Jinnah Sindh Medical University Karachi Pakistan

**Keywords:** case report, central venous catheter, end‐stage renal disease, foreign body retrieval, retained guidewire

## Abstract

Central venous catheterization (CVC) is a common procedure in clinical practice, yet retained guidewires represent a rare but potentially life‐threatening complication. Although most cases are recognized earlier, delayed detection in asymptomatic patients, especially those on hemodialysis, is rare and requires safe management protocols. A 45‐year‐old female with a 13‐year history of uncontrolled hypertension and end‐stage renal disease (ESRD) on long‐term hemodialysis was referred to the cardiology department following an incidental finding of a retained guidewire on a posterior–anterior (PA) chest X‐ray. The guidewire had been inadvertently left in situ during the placement of a permanent venous catheter in the right internal jugular vein at an outside hospital three months earlier. The lack of post‐catheterization imaging resulted in delayed recognition of the guidewire. Despite its prolonged retention, the patient remained asymptomatic and continued her routine hemodialysis sessions without access‐related complications. Imaging revealed a 50 cm J‐tip guidewire extending from the internal iliac vein to the right internal jugular vein. After obtaining informed consent, the guidewire was successfully retrieved using an endovascular approach in the interventional radiology suite. Post‐procedural imaging confirmed complete removal, and the patient was discharged in stable condition with no complications at follow‐up. This case highlights the importance of procedural vigilance, adherence to standardized protocols, and early intervention to mitigate the risks associated with retained intravascular foreign bodies.

**Key Clinical Message**

This case emphasizes procedural vigilance through strict adherence to standardized protocols, like guidewire counting, post‐procedure imaging, and early recognition of iatrogenic complications to prevent retained foreign bodies. It highlights the importance of minimally invasive management and aims to educate clinicians on meticulous techniques and prompt interventions in similar scenarios.

## 1 Introduction

Central venous catheterization (CVC) is an essential procedure in modern clinical practice, allowing for the administration of intravenous fluids, medications, blood products, parenteral nutrition, vasoactive agents, hemodialysis, and hemodynamic monitoring. CVCs are typically inserted into large central veins, most commonly the internal jugular, subclavian, or femoral veins, and advanced until the catheter tip resides within the superior vena cava, inferior vena cava, or right atrium [1, 2]. The technique most widely used for CVC insertion is the Seldinger technique, which, despite being generally safe and effective, carries inherent risks. Common complications include arterial puncture, pneumothorax, infection, and thrombosis.

A rare but potentially serious iatrogenic complication is the retention of the guidewire, either due to unrecognized failure to remove it or loss during the procedure [3, 4]. Guidewire retention is estimated to occur in approximately 0.02% (1:5000 cases) of CVC insertions [5, 6]. This complication can lead to serious sequelae such as cardiac arrhythmias, vascular injury, thrombosis, embolism, infection, cardiac perforation, or even tamponade [7]. Despite its rarity, guidewire retention remains a significant safety concern. Contributing factors include operator inexperience, lack of adherence to standardized protocols, and failure to confirm post‐procedure guidewire removal [2, 8, 9]. Efforts to reduce such adverse events have emphasized the use of procedural checklists, post‐procedure imaging, and team‐based verification of guidewire removal [10]. When guidewire retention is identified, endovascular retrieval is the preferred intervention due to its high success rate and minimally invasive nature. Techniques such as snare device capture are commonly employed in such cases [11].

Although guidewire retention is widely reported, its prolonged retention in asymptomatic cases, especially in hemodialysis‐dependent patients, is rare. This case describes a 45‐year‐old female with end‐stage renal disease (ESRD) who presented with an asymptomatic retained guidewire, discovered three months post‐catheterization. It emphasizes that even delayed recognition can be managed successfully with appropriate retrieval techniques and highlights the critical importance of procedural vigilance, adherence to standardized protocols, and the role of minimally invasive retrieval techniques in managing iatrogenic complications.

## 2 Case Presentation

A 45‐year‐old female with a 13‐year history of uncontrolled hypertension and ESRD on long‐term hemodialysis was referred to our department following an incidental finding of a retained guidewire visualized on a PA chest X‐ray. The guidewire had been inadvertently left in situ during the placement of a permanent venous catheter in the right internal jugular vein at an outside hospital three months prior. A post‐procedural X‐ray was not performed at that time, which led to delayed recognition of the guidewire, and despite its prolonged retention, the patient exhibited no signs of infection, thrombosis, or vascular compromise as she remained completely asymptomatic. She continued to undergo regular hemodialysis via the right internal jugular catheter and had no known prior medical history of vascular or catheter‐related complications. On examination, the patient appeared well, with stable vital signs and no tenderness, swelling, or erythema over the catheter insertion site.

**FIGURE 1:**
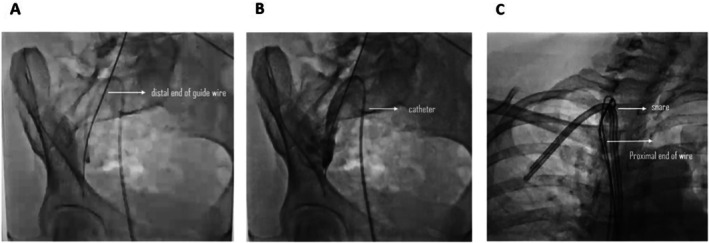
Fluoroscopic Imaging of Retained Guidewire. (A) The retained guidewire is visualized within the venous system, extending from the right internal jugular vein into the iliac vein. (B) Advancement of a balloon catheter within the venous system in an attempt to reposition or mobilize the retained guidewire. (C) Successful capture and withdrawal of the guidewire using an endovascular snare device.

A thorough imaging review confirmed a 50 cm J‐tip guidewire extending from the internal iliac vein to the right internal jugular vein (Figure 1). After obtaining informed consent, the patient was taken to the interventional radiology suite for endovascular retrieval of the retained guidewire. A 6 French femoral sheath was introduced into the right femoral vein using the Seldinger technique. A hydrophilic wire was advanced into the right internal iliac vein, followed by the insertion of a multi‐purpose catheter. Initial attempts to retrieve the distal portion of the guidewire using a snare were unsuccessful due to chronic endothelial embedding.

Efforts then shifted to the proximal portion of the guidewire within the right internal jugular vein. A hydrophilic wire was advanced to the subclavian vein, followed by a multi‐purpose catheter. After multiple attempts, the guidewire was successfully captured using a snare device and smoothly retrieved. Post‐procedure imaging confirmed the complete removal of the guidewire, with no remnant fragments visualized. The femoral sheath was removed, and hemostasis was achieved at the puncture site. The patient tolerated the procedure well and was transferred to the coronary care unit (CCU) for overnight observation. She was discharged in stable condition the following day.

At the one‐week follow‐up, the patient reported no complications. A follow‐up chest X‐ray confirmed the absence of any retained foreign bodies. She remained asymptomatic and resumed regular hemodialysis sessions without complications.

## 3 Discussion

The Seldinger technique of placement of a central venous catheter involves insertion of an introducer needle into the vein, advancing the guidewire through the needle, removing the needle, and then advancing the catheter over the guidewire. Once the catheter is in place, the guidewire is removed. However, a rare but potentially hazardous iatrogenic complication of this procedure is the retention of a guidewire, which can lead to dysrhythmia, vascular damage, thromboembolism, infection, cardiac perforation, and tamponade. In some cases, prolonged retention may lead to chronic complications such as venous stenosis, which can compromise vascular access, particularly in hemodialysis‐dependent patients [12]. However, it is usually asymptomatic, as in this case. It can be found incidentally, even several months after the procedure, on an X‐ray done for some other reason [13, 14]. Retained guidewires can result from operator inexperience, procedural distractions, failure to follow standardized protocols, and a lack of post‐procedure confirmatory imaging [15]. While the complication rate of inserting CVC catheters is approximately 15%, the intravascular loss of the guidewire during CVC placement is a rare but serious complication with reports of fatalities in up to 20% of the cases when the complete wire is lost [16, 17]. The failure to adhere to good procedural practices likely resulted in this complication. We strongly feel that this complication is preventable by following standard procedural protocol and by documenting the removal of the guidewire after CVC insertion [18]. Lastly, a post‐procedural chest x‐ray following CVC is always a good practice to avoid missed guidewire retention and early detection of such preventable complications. Globally, similar cases have been reported with varied outcomes. Symptomatic patients have required surgical retrieval, sometimes accompanied by minor complications or prolonged hospitalization [14, 19]. In contrast, asymptomatic cases that were managed with endovascular techniques showed variable success, with occasional retention of wire fragments [20]. These cases underscore the clinical spectrum of guidewire retention, influenced by both time to diagnosis and available intervention modalities. Compared to these, our patient had a similar delay in diagnosis (3 months) but differed as she remained completely asymptomatic. The successful retrieval without any complication or hospitalization beyond 24 h demonstrates that even prolonged retention can be safely managed with minimally invasive techniques when identified early and handled promptly.

Endovascular retrieval remains the gold standard for removing intravascular foreign bodies due to its high success rate and minimally invasive nature. The percutaneous retrieval of intravascular foreign bodies was first described in 1964. There are numerous techniques described in the literature. The majority of these techniques involve a Gooseneck snare, a Dormia basket, the two‐wire technique, 6‐F biopsy forceps, or even surgical intervention [21, 22]. Today, the most commonly used retrieval technique involves the use of snares. Studies have reported technical success rates exceeding 79% for percutaneous retrieval procedures with minimal complications and 96.6% in children [23, 24]. In cases where the guidewire is embedded within the endothelium, multiple retrieval attempts using different access points may be necessary, as demonstrated in this case.

This case highlights the importance of procedural vigilance and adherence to best practices in CVC placement to prevent retained foreign bodies. The successful endovascular retrieval of a chronically retained guidewire in an asymptomatic patient further underscores the efficacy of minimally invasive techniques in managing iatrogenic complications. Raising awareness about this preventable error through case reporting can contribute to improved clinical practices and patient safety outcomes.

## Author Contributions

**Abdul Qadir Nawabi:** formal analysis, project administration, supervision, validation, visualization, writing – original draft, writing – review and editing. **Noor Ahmad Jamal:** supervision, writing – original draft, writing – review and editing. **Wahidullah Lalzada:** data curation, resources, software, visualization, writing – review and editing. **Ahmad Jamshed Rezaie:** data curation, project administration, resources, supervision, validation, writing – review and editing. **Aamna Israr:** investigation, validation, writing – review and editing.

## Funding

No specific funding was received for this work. The research was performed as part of the employment and school course of the authors.

## Ethics Statement

This manuscript does not include personal or medical details about any identifiable individual. Written consent was obtained from the patient as per the journal's patient consent guidelines.

## Consent

Written informed consent was obtained from the patient for the publication of this case report.

## Conflicts of Interest

The authors declare no conflicts of interest.

## Data Availability

The data that supports the findings of this case report are available within the article. No additional data are available.
